# SMAD4 Y353C promotes the progression of PDAC

**DOI:** 10.1186/s12885-019-6251-7

**Published:** 2019-11-04

**Authors:** Zusen Wang, Yongxing Li, Shixiong Zhan, Lu Zhang, Shun Zhang, Qian Tang, Miaomiao Li, Zhen Tan, Shiguo Liu, Xiaoming Xing

**Affiliations:** 1 Department of Hepatobiliary and Pancreatic Surgery, Affiliated Hospital of Qingdao University, Qingdao, China; 2 Department of Pathology, Affiliated Hospital of Qingdao University, Qingdao, China; 3 Prenatal Diagnosis Center, Affiliated Hospital of Qingdao University, Qingdao, China

**Keywords:** Pancreatic ductal adenocarcinoma, SMAD4, Mutation, E-cadherin, Vimentin

## Abstract

**Background:**

SMAD4 is frequently inactivated and associated with a poor prognosis in pancreatic ductal adenocarcinoma (PDAC). Abnormal SMAD4 expression also plays an important role in the malignant progression of PDAC.

**Methods:**

We investigated SMAD4 status in PDAC by immunohistochemical methods to explore the relationships between SMAD4 expression and clinicopathological features and then detected SMAD4 mutations by Sanger sequencing in 95 patients with PDAC to identify new mutation sites in PDAC. We further evaluated the effects of a missense mutation, Y353C, in the SMAD4 MH2 domain, on cell proliferation and migration in vitro.

**Results:**

Immunohistochemistry showed that the expression of SMAD4 in PDAC carcinoma tissue was significantly lower than that in normal pancreatic tissue, and negative SMAD4 expression was closely related to tumour diameter, staging, lymph node metastasis and differentiation*.* Sanger sequencing analysis showed that the rate of SMAD4 mutation was 11.8% in 85 PDAC cases, and the novel SMAD4 Y353C missense mutation identified in this study promoted cell migration and invasion without affecting cell proliferation in vitro*.* Furthermore, SMAD4 Y353C resulted in reduced expression of E-cadherin and increased expression of Vimentin compared with wild-type SMAD4 overexpression.

**Conclusion:**

This study supports the key role of SMAD4 as a tumour suppressor gene in PDAC and shows that SMAD4 Y353C is associated with poor progression of PDAC.

## Background

Pancreatic ductal adenocarcinoma (PDAC) is a devastating cancer in that most patients are diagnosed at an advanced clinical stage, and treatment is difficult because only 15 to 20% of patients can be accepted for surgical treatment [1]. The median overall survival of PDAC patients is less than 7 months [2]; morbidity exceeds 4% [3]; and the 5-year survival rate is constant at 6% [4–6]. PDAC is predicted to be the second leading cause of cancer-related deaths by 2020 in the USA [7], seriously threatening public health. As the absence of biomarkers for early detection delays the diagnosis and treatment of PDAC, the search for appropriate tumour biomarkers for patients with PDAC is extremely urgent.

It has been reported that pancreatic carcinoma involves an average of 63 genetic alterations mainly related to 12 cellular signalling pathways [8] and that the genetic lesions arise through activating mutations of *KRAS* and inactivation of the *INK4A*, p53-ARF and SMAD4 pathways [9–11]. The tumour suppressor gene SMAD4, originally detected on human chromosome 18q21.1, is commonly referred to as pancreatic cancer deletion gene4 (DPC4) because deficiency in its expression was first found in pancreatic cancer [12]. In 1996, Hanh et al. [13, 14] found that SMAD4 deletion or mutation caused a loss of expression in 50% of pancreatic cancers. Recently, numerous studies have reported that SMAD4 is genetically inactivated in different patterns between human populations in different regions, with the inactivation rates of SMAD4 in western countries being higher than those in Asia [10, 13, 15–17]. Low expression of SMAD4 is correlated with poor outcomes and serves as a prognostic factor [18–20]. However, there have been conflicting results indicating that SMAD4 expression is not associated with a poor prognosis in patients with pancreatic cancer [21–23].

SMAD4 is a mediator that was first studied in the context of TGF-β family signal transduction; SMAD4 contributes to signal transfer from the membrane to the nucleus and directly affects downstream responses [24]. Previous studies have shown that SMAD4 also inhibits cell migration [25, 26] and that its downregulation affects tumour cell proliferation [27]. However, there are controversial reports indicating that SMAD4 can accelerate migration [28]. Therefore, we conducted a retrospective study to estimate the relationship of SMAD4 expression with clinical characteristics and overall survival (OS) in 95 Chinese Han PDAC patients. Additionally, all exons of SMAD4 were screened for mutations, and the potential effects of a missense mutation occurring in the SMAD4 MH2 domain on cell proliferation, migration, invasion, and epithelial-mesenchymal-transition (EMT) in the SW1990 and PANC-1 cell lines were determined.

## Methods

### Tissue collection

We collected a cohort of 95 paraffin-embedded specimens from patients with resectable primary PDAC who underwent pancreaticoduodenectomy at the Affiliated Hospital of Qingdao University from January 2011 to July 2017. To avoid false negatives from pancreatic ductal adenocarcinoma tissue with fibrous tissue, we chose specimens accounting for more than 60% of the total area and removed as much interstitial tissue as possible. The pathological TNM stage standard was applied according to the American Joint Committee on Cancer (AJCC) Eighth Edition. The main results of this study were the overall survival period determined from the time of Whipple resection to the date of death or the last follow-up, where the final observation was recorded in December 2017.

### Immunohistochemistry

The collected tissue paraffin blocks were sectioned, stained with H&E, and re-diagnosed by pathologists, and typical lesions and adjacent tissue were labelled. A tissue chip maker was used to punch holes (2.0 mm in diameter) in blank paraffin blocks. According to the location of the lesions marked in the H&E slices, the same diameter of tissue was obtained from the corresponding position of the tissue wax block, and the tissue number was recorded. Because of the obvious proliferation of interstitial tissue in pancreatic ductal adenocarcinoma tissue, we performed immunohistochemistry on the entire sections of 10 specimens that could not be perforated. Paraffin sections were deparaffinized and rehydrated for antigen retrieval. All sections were incubated with an anti-SMAD4 monoclonal antibody (EP618Y, Abcam, UK) at a dilution of 1:200 for 60 min. Labelling for SMAD4 was carried out strictly according to the manufacturer’s protocol using the Envision Plus Detection Kit (DAKO, Carpinteria, CA), and nuclei were counterstained with haematoxylin. The assessment of immunohistochemical expression was performed in a blinded manner by two experienced pathological experts. Compared with the surrounding normal tissue, diffuse staining of SMAD4 was defined as positive expression in the tumour tissue, and no or little staining indicated negative expression [29].

### Sanger sequencing

Among the 95 specimens that we collected, 10 specimens with more than 40% interstitial components were removed, and the remaining 85 specimens were sequenced. The samples were cut into 5–8 slices with a 5–10 mm thickness, and whole-genome DNA was extracted using the TIANamp FFPE DNA Kit (TIANGEN Biotech, Beijing, China) according to the manufacturer’s instructions. The concentration of DNA was quantified with a Thermo Scientific Multiskan Go. Reaction system via PCR (20 μL), where each reaction contained 2× Master Mix (blue) (Tsingke Biotech, Beijing, China) (10 μL), primers (10 bp; 0.5 μL), DNA template (20~80 ng/mL; 2 μL), and distilled water (7 μL). The PCR parameters were as follows: pre-denaturation for 5 min at 94 °C; 35 cycles of denaturation at 94 °C for 30 s, annealing at 54~60 °C for 1 min, and extension at 72 °C for 45 s; and a final extension at 72 °C for 10 min (Additional file 1: Figure S1: primer details). PCR products (Additional file 1: Figure S2) were separated by 1.5% agarose gel electrophoresis and purified with a GeneJET Gel Extraction Kit (Thermo Scientific™ K0691). Sanger sequencing was carried out by Qingdao Tsingke Biological Technology.

### Sequence alignment

Sequence data were matched to the original gene sequence of SMAD4 (NM_005359.5) from NCBI GenBank (https://www.ncbi.nlm.nih.gov/) by using BioEdit and DNAMAN software.

### Expression vector and plasmid construction

We constructed negative control, SAMD4 wild-type and SAMD4 mutant expression vectors to investigate the effect of a missense mutation in the MH2 domain. The primers for the wild-type SMAD4 coding region were as follows: forward: AATTGCTTCAGAAATTGGAGACATATT, reverse: CAGGATTGTATTTTGTAGTCCACCAT, where amplification was performed by using the *TransStart*® *FastPfu* Fly DNA Polymerase kit (Transgen, Beijing, China), and the product was sequenced (Tsingke Biotech, Qingdao, China). Subsequently, we used a Fast Mutagenesis System Kit (Transgen, Beijing, China) to induce site-directed mutagenesis of the mutant type with the SMAD4 wild-type PCR product as the template, where the sequences of the primers were as follows: forward: 5′-TTACTGTTGATGGATGCGTGGACCCT-3′, and reverse: 5′-CATCCATCAACAGTAACAATAGGGC-3′. We constructed wild-type and mutagenesis-type expression vectors by using the *pEASY*®-Blunt M2 expression vector kit (Transgen, Beijing, China), and the *pEASY*®-Blunt M2 expression vector without SMAD4 served as a negative control. The plasmid DNA was extracted from cultured competent cells by overnight shaking (250 rpm) at 37 μC using the EndoFree Mini Plasmid Kit II (TianGen Biotech, Beijing, China) according to the manufacturer’s instructions.

### Cell culture and transfection

The SW1990 and PANC-1 human pancreatic cancer cell lines were kindly provided by Dr. Zhou Yanbin (Affiliated Hospital of Qingdao University, Qingdao, China) and cultured in RPMI 1640 (HyClone, Shanghai, China) and DMEM (HyClone, Shanghai, China) supplemented with 10% foetal bovine serum at 37 °C in a 5% CO_2_ atmosphere, respectively. Transfection was performed using the TransTntro™ EL Transfection Reagent (Transgen, Beijing, China) according to the manufacturer’s protocol and verified by real-time quantitative polymerase chain reaction (qRT-PCR) and western blotting.

### Western blotting

After 48 h of SW1990 and PANC-1 cell transfection, the cells (1 × 10^6^ cells) were washed with PBS two times and then lysed with 150 μL of RIPA lysis buffer with a 1% phenylmethanesulfonyl fluoride (PMSF) mixture for 30 min. Thereafter, the lysates were centrifuged at 4 °C at 12000 g for 15 min, and the supernatant was used. An enhanced BCA protein assay kit was used to detect the protein concentration (Beyotime, Shanghai, China). A 25 μg standard protein solution was diluted to 20 μL with PBS, and 5 μL of protein buffer was added to an OSE-DB-01 metal bath (TIANGEN Biotech, Beijing, China) for 10 min at 95 °C. The obtained protein samples were then loaded into a 10% SDS-PAGE separation gel to separate the total proteins, followed by transfer to a polyvinylidene fluoride (PVDF) membrane. Then, the membrane was sealed with 5% skimmed milk at room temperature for 1 h, followed by incubation overnight at 4 °C with the primary antibody at a concentration of 1:3000 for SMAD4 (EP618Y, Abcam, U.S.A.), 1:1500 for Vimentin (5G3F10, Santa Cruz, USA), 1:2000 for E-cadherin (G-10, Santa Cruz, USA) or 1:1000 for GAPDH (AB9483, Abcam, U.S.A.). The appropriate secondary antibody coupled to horseradish peroxidase (HRP) was then applied to the membrane at a concentration of 1:4000, followed by incubation at room temperature for 1 h. The protein bands were detected by enhanced chemiluminescence (Thermo Fisher Scientific, USA).

### RNA extraction, cDNA synthesis and real-time quantitative polymerase chain reaction (qRT-PCR)

Total RNA was isolated after 24 h of SW1990 and PANC-1 cell transfection using TRIzol (Invitrogen, Carlsbad, U.S.A.) following the manufacturer’s instructions. 1 μL of total RNA was used for cDNA synthesis with TransScript One-Step gDNA Removal and cDNA Synthesis SuperMix (Transgen, Beijing, China). A quantitative reverse-transcriptase polymerase chain reaction (qRT-PCR) kit (Transgen, Beijing, China), the corresponding gene-specific primers and the TIB8600 Real-Time PCR System (Triplex International Biosciences, China) were used to amplify cDNA. Primer synthesis was completed by Sangon Biotech (China). The primer sequences were as follows: SMAD4: forward: 5′-CCTTCAAGCTGCCCTATTGTTACT-3′, reverse: 5′- ACATTCCAACTGCACACCTTTG-3′; E-cadherin: forward: 5-TGGATAACCAGAATAAAGACCAAGTG-3, reverse: 5- TCCTCCGAAGAAACAGCAAGA-3′; Vimentin: forward: 5′-GCAGGAGGCAGAAGAATGGTA-3′, reverse: 5-GGGACTCATTGGTTCCTTTAAGG-3′; GAPDH: forward: 5′- CTGACTTCAACAGCGACACC-3′, reverse 5′-TGCTGTAGCCAAATTCGTTGT-3′. The reaction conditions for qRT-PCR were as follows: pre-denaturation at 94 °C for 30 s, followed by 40 cycles of amplification at 95 °C for 5 s, 60 °C for 15 s and 72 °C for 10 s; the CQ value was then calculated, and each sample was run in triplicate. After each PCR run was complete, the product melting curve was analysed to confirm the presence of a single product and that the data could therefore be used. The data were standardized with GAPDH as the internal reference, and the expression levels of the target genes were analysed using the comparative threshold cycle method (2^−ΔΔCT^).

### Cell proliferation assay

SW1990 and PANC-1 cells were seeded in 96-well plates at an inoculation density of 0.5 × 10^5^/well, and each group included 8 side wells. After 24 h, these cells were transfected with 0.2 μg/ml of the M2 expression plasmid negative control vector, SMAD4 wild-type *pEASY*®-Blunt M2, or SMAD4 mutant-type *pEASY*®-Blunt M2, and grown for another 24 h, 48 h, or 72 h. Thereafter, 10 μl or 90 μl of the CCK reagent in conventional culture medium was added to each well, followed by incubation for 1 h, and the OD value of enzyme at 450 nm was detected in a microplate reader (Thermo Fisher Scientific, USA).

### Wound healing assay

Monolayers of cells that were cultured in 6-well plates for approximately 24 h were washed with PBS twice and scratched with a pipette tip, then cultured in medium without foetal bovine serum for 24 h. The cells were subsequently photographed under a microscope, and cell migration distances were calculated.

### Transwell assay

Cell Transwell assays were performed using a two-chamber migration setup with 6.5 mm-diameter insert with an 8-μm pore size (Corning Incorporated, Corning, NY, USA). Monolayer cells were uniformly plated in 24-well plates and then incubated until 60–70% confluence and transfected with the plasmids. These cells were then incubated for another 12 h. Trypsinized cells were resuspended in PBS twice, after which the PBS was removed, and 400 μL of serum-free medium was added following centrifugation. The suspension cells was again diluted, homogenized to obtain a single-cell suspension, and counted in a cell counting plate to calculate the cell concentration. A total of 1 × 10^4^ pancreatic cancer cells were seeded into the upper chambers, and DMEM /RPMI 1640 supplemented with 20% FBS was used to fill the lower compartment. Following 12 h of incubation at 37 °C in a humidified atmosphere containing 5% CO_2_, the cells remaining in the upper chamber were removed with a cotton swab. The filters were fixed in absolute methanol at 37 °C for 30 min and stained with 0.05% crystal violet at 37 °C for 30 min. Then, the crystal violet dye was washed away with PBS (1×). The cells that migrated to the lower surface were counted under a microscope (20×). For the invasion assay, the bottom surface of each membrane was pre-coated with 40 μL of cold Matrigel (BD Bioscience, Franklin Lakes, NJ, USA; diluted 1:1). Cells at the bottom of the wells were quantified after 48 h of incubation at 37 °C using a light microscope. Cells that migrated to the bottom surface of the membrane were observed under light microscopy and then photographed under magnification (20×). Each independent experiment was repeated at least three times.

### Statistical analysis

Statistical analysis of the data was performed using SPSS software 20.0 (IBM Corporation, Chicago, U.S.A.). Chi-square tests were applied for statistical assessment of the relationship between clinicopathological characteristics. Survival analysis was performed using the Kaplan-Meier method; survival curves were drawn; and the survival rates of the two groups were compared using the log-rank test. The comparisons of the differences between groups were statistically analysed by one-way ANOVA. *P < 0.05* was considered statistically significant.

## Results

### Patients

Most of the cases included in this study were stage I~II A (67/95), and the rest were stage II B or IV (28/95), with no stage III cases included. This study was approved by the ethics committee of the Affiliated Hospital of Qingdao University, and informed consent was obtained from all patients. The details of patients will not be disclosed.

### SMAD4 expression analysis

**Fig. 1:**
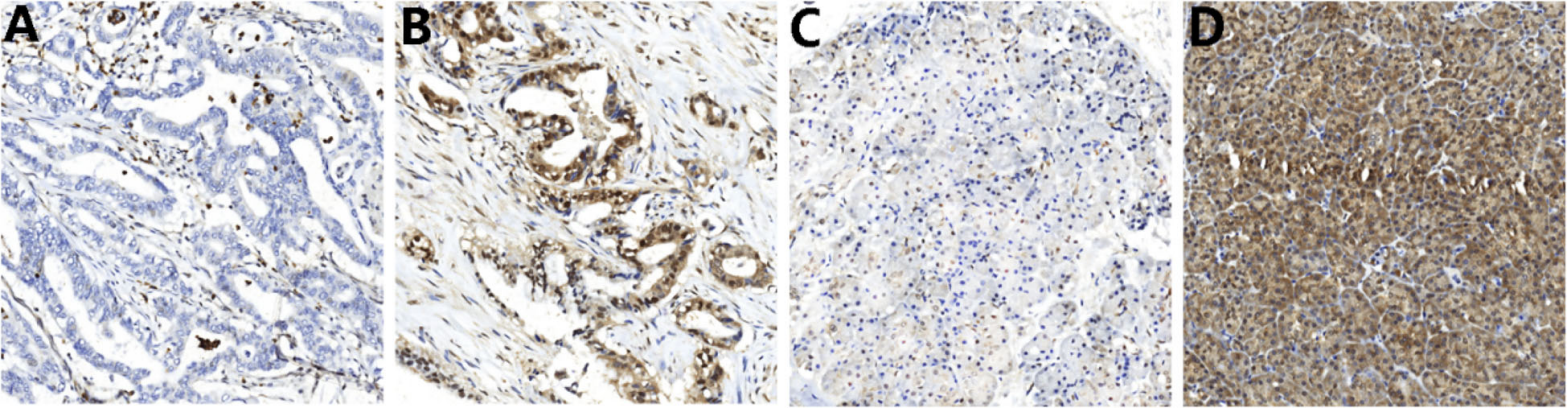
SMAD4 expression status in pancreatic ductal adenocarcinoma. Representative images of negative (**a**) and positive (**b**) expression of SMAD4 in carcinoma tissues. Representative images of negative (**c**) and positive (**d**) expression of SMAD4 in adjacent tissues

SMAD4 presented loss of 25% (21/95) in normal pancreatic tissues and 75.7% (71/95) in carcinoma tissues (Table 1). Most of the PDAC tumour tissues were stained with SMAD4 in the cytoplasm, and some of them exhibited expression in the interstitial fibrous tissue of the tumour (Fig. 1). The chi-square test showed a significant difference in the expression of SMAD4 between the cancer and adjacent tissues (χ2 = 48.511, *P < 0.001*).

**Table 1:** SMAD4 expression in cancer tissues and adjacent tissues

Group	SMAD4 expression		χ^2^	*p*-value
	Intact SMAD4	Lost SMAD4		
Tumor tissue	23 (24.5%)	72 (75%)	48.511	<0.001
xAdjacent tissue	71 (75.7%)	24 (25%)

### SMAD4 expression is associated with the clinicopathologic features of PDAC but not the overall survival rate

**Fig. 2:**
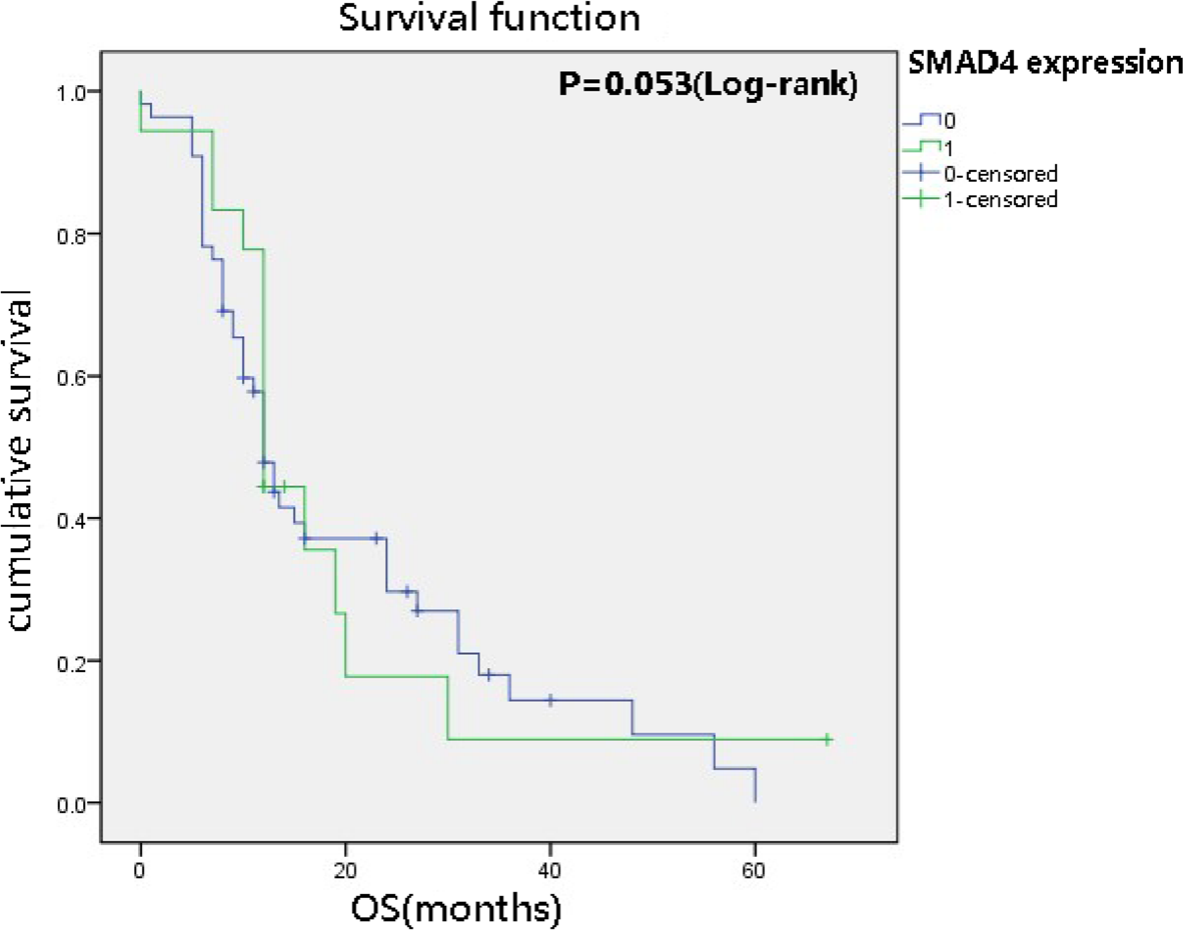
Univariate analysis (Kaplan–Meier curves and log-rank tests) of SMAD4 expression status and OS in 73 patients. The loss of SMAD4 expression was not unrelated to the overall survival rate (*P>0.05*, no statistically significant difference)

We analysed the relationships between SMAD4 expression and the clinicopathological factors as well as the overall survival rate of PDAC. Negative SMAD4 expression showed a significant relationship with tumour diameter (χ^2^ = 6.389, *P* = 0.016), TNM staging (χ^2^ = 6.303, *p* = 0.016), lymphatic invasion (χ^2^ = 74.451, *P* = 0.000) and differentiation (χ^2^ = 5.272, *P* = 0.039). However, there was no correlation with sex, age, tumour location or perineural invasion (Table 2). Twenty-two patients were lost to follow-up after surgical treatment, and the follow-up rate was 76.8%. Thus, survival analyses of patient outcomes were performed for this cohort of 73 patients. The median survival time of the PDAC group was 12 months, and the median survival time of the SMAD4 deletion and expression groups was also 12 months (Fig. 2). The loss of SMAD4 expression was not related to the overall survival rate (OS) (*P = 0.053*).

**Table 2:** Relationship between SMAD4 expression and clinic pathological features

Group	SMAD4 expression		χ^2^	*p*-value
	Intact SMAD4	Lost SMAD4		
Sex				
Male	8 (34.78%)	25 (34.7%)	<0.001	1.000
Female	15 (65.22%)	47 (65.3)		
Age (year)				
<60	11 (47.8%)	19 (26.4%)	3.708	0.072
≥60	12 (52.2%)	53 (73.6%)		
Tumor diameter (cm)				
<3	11 (47.8%)	15 (20.8%)	6.389	**0.016**
≥3	12 (52.2%)	57 (79.2%)		
TNM tumor staging				
IA~IIA	21 (91.3%)	46 (63.9%)	6.303	**0.016**
IIB~IV	2 (8.7%)	26 (36.1%)		
Lymphatic invasion				
Yes	2 (8.7%)	70 (97.2%)	74.451	**<0.001**
No	21 (91.3%)	2 (2.8%)		
Tumor location				
Head	11 (47.8%)	47 (65.3%)	2.233	0.149
Body and tail	12 (52.2%)	25 (34.7%)		
Differentiation				
High and middle differentiation	12 (52.2%)	19 (26.4%)	5.272	**0.039**
Poorly differeftiation	11 (47.8%)	53 (73.6%)		
Perineural invasion				
Yes	9 (39.1%)	22 (30.6%)	0.583	0.610
No	14 (60.9%)	50 (69.4%)		

The bold values indicate *P* values less than 0.05

### Analysis of sanger sequencing results for SMAD4

In the 85 PDAC cases, the mutation rate was 11.8%, including point mutations, base insertions and deletions (Table 3, Additional file 1: Figure S3). In this study, we found 4 novel heterozygous missense mutations in exons 1, 3, and 8 [c.29C>T (pP10L), c.34A>G (pS12G), c.112A>G (p. R38G) and c.1058A>G (pY353C)] and 2 synonymous mutations in exon 1 [c.201 T>C (p.H67=), c.6C>T(p.D2=)]. The comparison of SMAD4 protein sequences between 6 species (*Mus musculus, Homo sapiens, Macaca mulatta, Pan troglodytes, Monopterus albus* and *Schistosoma mansoni*) with DNAMAN indicated that p. S12G, p. R38G and p. Y353C were conserved, among which PY353C located in the MH2 region played a significant role in forming oligomers with other SMAD4 molecules and regulating transcriptional function. Therefore, we further elucidated the functional changes caused by SMAD4 Y353C in our subsequent work. In addition, we detected 3 heterozygous nucleotide deletions, which occurred in exons 1, 2, and 8 (c.153_delA, c.352_363delGCGTTTGACTTA, c.1242-1245delAGAC) as well as a one-nucleotide insertion in exon 8 (c.1103_1104insG)*.* The deletions and insertions usually caused a base shift, which resulted in an early termination codon and suppressed protein expression.

**Table 3:** SMAD4 mutation sites in PDAC

Exon	Mutation type	Nucleotide change	Mutation results	Probably damaging
1	Synonymous	c.6C>T	p.D2D=	**–**
1	Missense	c.29C>T	p.P10L	0.819
1	Missense	c.34A>G	p.S12G	0.748
1	Missense	c.112A>G	p.R38G	1
1	deletion	c.153_delA	Frameshift, stop at codon 57(TAA)	**–**
1	Synonymous	c.201 T>C	H67H=	**–**
2	deletion	c.352_363delGCGTTTGACTTA	A118_L121del, Frameshift	**–**
8	Missense	c.1058A>G	p. Y353C	1
8	Insertions	c.1103_1104insG	Frameshift and stop at codon 377(TGA)	
9	deletion	C.1242-1245delAGAC	L414_D415del, frameshift and stop at codon 434(TAA)	**–**

### The SMAD4 Y353C mutation has a negative effect on the expression of SMAD4 in vitro

**Fig. 3:**
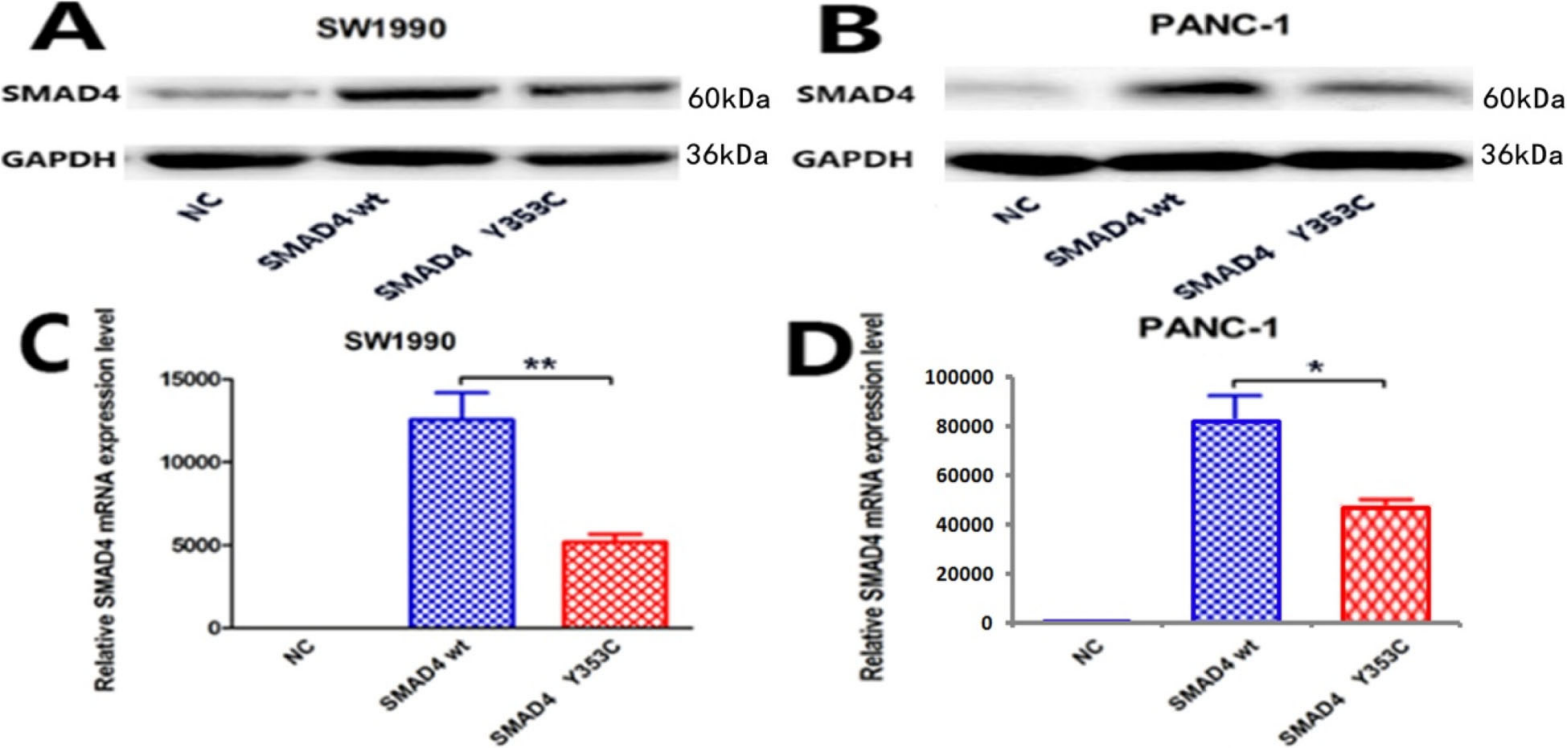
Protein and mRNA expression of SMAD4 after the transfection of cells with plasmids. **a**, **b** Representative western blot images of SW1990 and PANC-1 cells. GAPDH was included as a loading control. **c**, **d** The mRNA expression levels of the SW1990 and PANC-1 cells determined by qRT-PCR showed the same trend as the western blot results. (* indicates a significant difference by one-way ANOVA, *P < 0.05, **P < 0.001)

qRT-PCR and western blot analyses revealed that negative control-transduced SW1900 and PANC-1 cells showed the lowest SMAD4 expression at both the mRNA and protein levels. Western blot analysis and qRT-PCR indicated that SMAD4 was successfully overexpressed in human pancreatic cancer cells compared to negative control cells. SMAD4 Y353C-transduced expression in the SW1990 and PANC-1 cell lines was lower than that in the wild-type-transduced cells (Fig. 3).

### The SMAD4 Y353C mutation has no effect on cell proliferation in vitro

To investigate whether the SMAD4 Y353C mutation affects cell proliferation, we detected the proliferation rate of transfected SW1990 and PANC-1 cells at different time points. The results showed that there was no statistically significant difference in cell proliferation (Additional file 1: Figure S4).

### The SMAD4 Y353C mutation increases cell migration and invasion in vitro

**Fig. 4:**
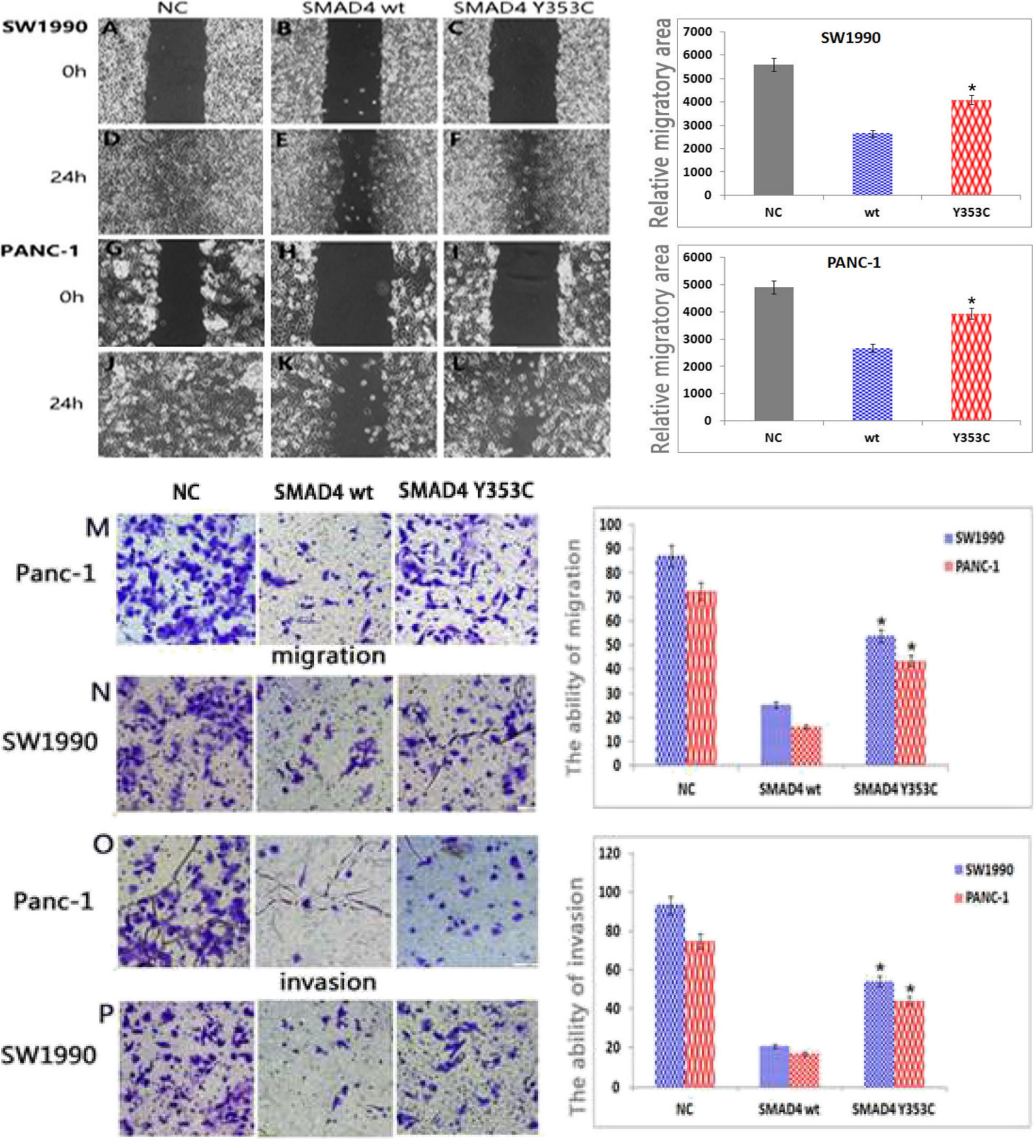
SMAD4 Y353C attenuates the inhibitory effect of SMAD4 on cell migration and invasion. The images of SW1990 (**a**~**f**) and PANC-1 (**g**~**l**) cells are representative of three independent experiments (magnification 40×). The relative migratory area is shown on the right. Migration (**m**, **n**) and invasion (**o**, **p**) were determined in Transwell assays of SW1990 and PANC-1 cells. The results of the quantification of cell migration and invasion are shown (right). All the statistical data were analysed by one-way ANOVA. All data are shown as the mean ± SD of 3 independent experiments performed in triplicate (*P < 0.05 vs SMAD4 wt or NC)

To explore the effect of SMAD4 Y353C expression on the migratory and invasion potential of SW1990 and PANC-1 cells in vitro, wound healing and Transwell assays were performed. The results indicated that wild-type SMAD4 overexpression inhibited cell migration compared with the negative control cells. In addition, SMAD4 Y353C increased cell migration ability compared to the SMAD4 wild-type cells in vitro (Fig. 4a-l). Moreover, we found that SMAD4 Y353C promoted cell invasion ability compared to the SMAD4 wild-type cells in Transwell assays (Fig. 4m-p). Overall, these data showed that SMAD4 Y353C weakened the ability of SMAD4 to suppress migration and invasion in PANC-1 and SW1990 cells in vitro.

### SMAD4 Y353C promotes EMT in pancreatic cancer

**Fig. 5:**
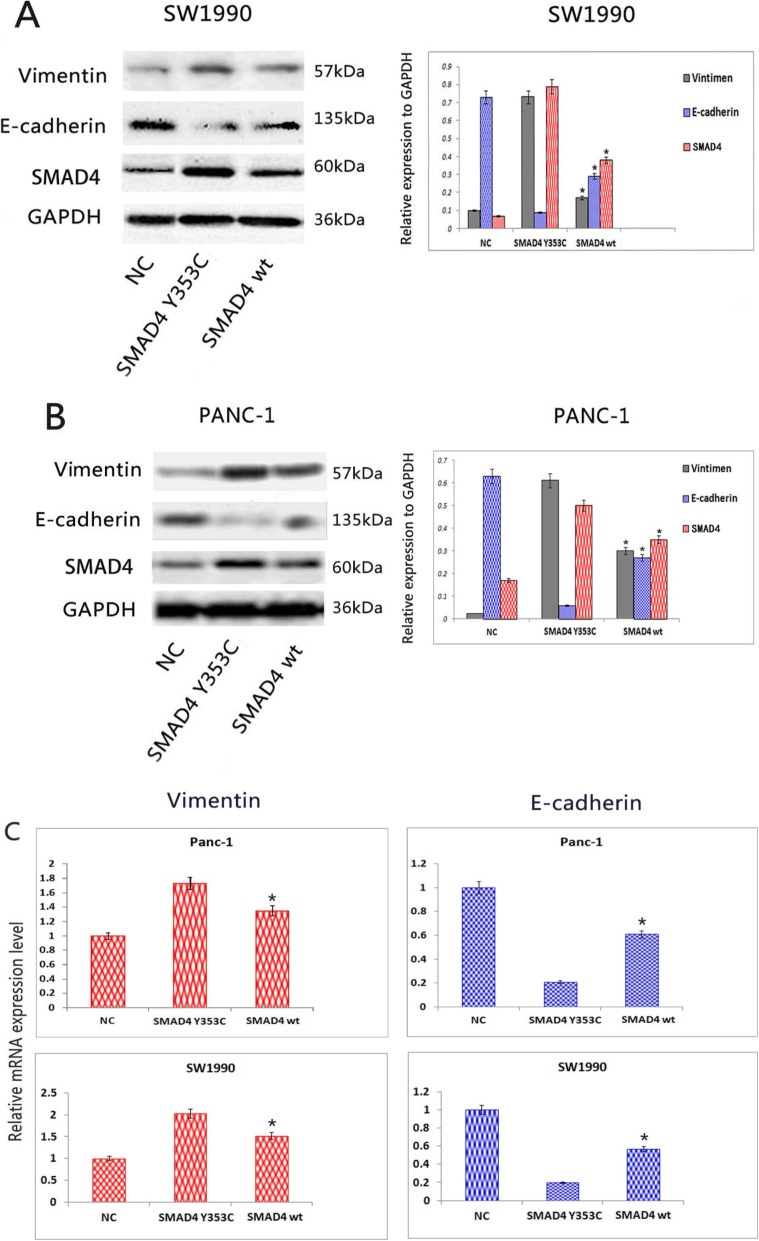
SMAD4 Y353C reduces the expression of E-cadherin but promotes that of Vimentin. **a**, **b** Western blot analysis of EMT markers was performed in the SW1990 and Panc-1 cell lines. GAPDH was included as a loading control. **c** The relative mRNA levels of EMT markers were examined by qRT-PCR. All statistical data were analysed by one-way ANOVA. Three independent experiments were conducted for each assay (*P < 0.05 vs SMAD4 wt)

EMT plays a vital role in pancreatic carcinoma invasion and distant metastasis. Cancer cells lose related epithelial characteristics and gain invasion ability during the process of EMT [30]. Hence, we further investigated whether the mutation of the SMAD4 protein influenced EMT markers such as E-cadherin and Vimentin through western blotting (Fig. 5a, b). Our experiment showed that SMAD4 Y353C inhibited the expression of the E-cadherin protein but increased the expression of Vimentin compared with the SMAD4 wild-type cell lines. We obtained the same results through the analysis of mRNA levels by qRT-PCR (Fig. 5c).

## Discussion

In the present study, we showed that the expression of SMAD4 was significantly lower in pancreatic cancer tissues than in adjacent tissues by analysing 95 paraffin-embedded tissue sections of pancreatic cancer. We also found that the SMAD4 mutation rate reached 11.8% according to Sanger sequencing and revealed 4 novel mutations. The novel mutation Y353C, located in the MH2 region, which plays an important role in regulating transcriptional function, was further confirmed to contribute to the low expression of SMAD4 and to significantly alter the bioactivity of the SW1990 and PANC-1 cell lines. This basic research provides powerful theoretical support for the targeted treatment of pancreatic cancer.

SMAD4 is a vital molecule in the TGF-β pathway, which has broad effects on tumour pathology. The SMAD proteins are classified into three categories: receptor-regulated SMADs, co-mediated SMADs and inhibitory SMADs. The SMAD4 protein is the sole member of the co-mediated SMAD protein group [31]. SMAD4 binds to receptor-regulated SMADs (SMAD2 and SMAD3) when it is activated by TGF-β upstream signals, then forms an oligomer complex and enters the nucleus to regulate the expression of target genes [32, 33]. The aberrant expression of SMAD4, including genetic alterations or homozygous deletion, can directly alter the normal signalling of the TGF-β pathway and lead to uncontrolled cell growth and tumour induction in the pancreas [34]. In our study, loss of SMAD4 was observed in 75% (72/95) of the examined samples, which was similar to the reported incidence of 32–81.6% [11, 34–36]. Additionally, we found that the loss of SMAD4 expression was positively correlated with a large tumour diameter (≥ 3 cm), the number of patients with TNM tumour staging (IA~IIA and IIB~IV), lymphatic invasion and poor differentiation, suggesting that the negative expression of SMAD4 in these 95 cases was related to the degree of PDAC malignancy. According to our findings, the loss of SMAD4 expression was irrelevant to the overall survival rate. However, Singh’s group found that the median survival was 5 months in a group showing SMAD4 alterations and 10 months in a wild-type SMAD4 group; these authors focused on an Indian population with PDAC and examined SMAD4 alterations and the effect of SMAD4 loss on patient survival through the analysis of 249 PDAC cases [34]. Other researchers have reported similar findings based on extensive follow-up [37–40]. We suspect that our negative result is mainly related to our small sample size, which directly affects the data statistics. The sample collection area should be considered too small. Expanding the sample size and collecting sample data from different regions will make the results more convincing, which will be our next research goal.

In our further analyses, we found that the mutation rate of SMAD4 in the 85 cases was 11.8%, which was lower than that previously reported in PDAC, ranging from 20 to 55% [9, 13, 41]. Recently, Hayashi et al. [10] performed a targeted deep sequencing assay in 100 surgically resected pancreatic carcinoma cases from Japan and found that the rate of mutations in SMAD4 was approximately 13%, which was consistent with the results of our study. Some studies have suggested that SMAD4 mutational hotspots are located in exons 8 and 11 and that a mutational cluster exists in the MH2 domain [14]. Our sequencing results showed that among the 10 total mutations identified, 5 point mutations occurred in exon 1, and only 2 were found in the hotspot in exon 8, while we did not find any mutations in exon 11. Some studies have shown that the prognosis of pancreatic cancer with a SMAD4 mutation in the MH2 domain is poor [34]. Therefore, we undertook further analyses to determine the effect of SMAD4 Y353C located in the MH2 region on pancreatic cancer cells and revealed its important role among 4 novel heterozygous missense mutations.

We found that SMAD4 Y353C did not show effects on cell proliferation but promoted cell migration and invasion compared with the wild type. Chen et al. [28] indicated that SMAD4 re-expression or knockdown did not significantly affect tumour growth in vitro and in vivo but that wild-type SMAD4 reduced cell migration and invasion through up-regulation of the activation of the SMAD4-dependent TGF-β pathway. These results suggested that the expression of SMAD4 is closely related to the malignancy and metastasis of pancreatic cancer cells.

EMT is closely related to the invasion and metastasis of various types of tumours and is positively correlated with a poor prognosis [42–44]. Moreover, similar research revealed higher expression of EMT-induced markers such as Vimentin and lower expression of E-cadherin in pancreatic cancer [45, 46]. Therefore, we performed associated assays and obtained similar results to other reports indicating that SMAD4 Y353C induces low expression of E-cadherin and significantly increases Vimentin levels compared to wild-type SMAD4-. Many researchers have found that cells induced to undergo EMT can gain cancer stem cell (CSC) properties, with the enrichment of a CD44(+)/CD24(−) subpopulation, which is responsible for tumour initiation and formation [47–49]. Based on this information, we speculate that SMAD4 Y353C may induce the malignant transformation and metastasis of cancer cells by regulating CSCs, which should be further investigated in the future.

In addition to the classical TGF-β/SMAD4 signalling pathway, other pathways are also closely related to ectopic SMAD4 in the development of pancreatic carcinoma. Previous studies [50–52] have indicated that the oncogenic KRAS pathway activates mutation and promotes negative SMAD4 expression, which in turn activates the ERK/MAPK signalling pathway to induce the motility, migration and invasion of malignant cells. KRAS can also promote tumour metastasis and EMT progression by inhibiting RKIP in conjunction with MAPK-ERK signalling [53, 54]. In the JNK pathway, SMAD4 inhibits JNK activity by increasing MKP1 levels to reduce VEGF expression, thus attenuating the development of pancreatic cancer cells [55]. In addition, non-coding RNA plays a vital role in the SAMD4-mediated pathway. For example, a low level of microRNA-494 contributes to increasing the transcriptional activity of β-catenin and promoting the expression of Wnt, leading to pancreatic tumourigenesis via the regulation of stem-cell renewal and induction of EMT in SMAD4-deficient pancreatic cancer cells [56, 57]. Therefore, the absence of SMAD4 always favours the induction of cancerous transformation and distant metastasis, but the comprehensive network of molecules associated with SMAD4 remains unclear. Therefore, it is necessary for us to conduct further in-depth research on SMAD4 and its mutation-related mechanisms in pancreatic cancer.

## Conclusion

In summary, the negative expression of SMAD4 was found to be related to malignant phenotypes of PDAC such as lymph node metastasis, tumour size and the degree of differentiation. This study indicated that SMAD4 overexpression inhibits migration and invasion and that this inhibitory effect is weakened in SMAD4 Y353C cells. Additionally, we revealed that SMAD4 Y353C reduces the expression of E-cadherin and promotes Vimentin expression. We will expand the original sample size to multiple locations to rigorously demonstrate the relationship between overall survival and SMAD4 Y353C. It is still necessary to further explore the mechanism whereby SMAD4 Y353C acts as a tumour suppressor gene and, thus, provide a firmer theoretical basis for PDAC treatment.

## Supplementary information

**Additional file 1: Figure S1.** The whole exons of SMAD4 gene primers for details. **Figure S2.** SMAD4 gene 1~11 exons’ PCR products electrophoresis results. **Figure S3.** SMAD4 mutation analysis. (A) c.6C>T (p.D2D=); (B) c.29C>T (p.P10L); (C) c.34A>G (p.S12G); (D) c.112A>G (p.R38G); (E) c.153_d3elA [Frameshift, stop at codon 57(TAA)]; (F) c.201 T>C (p.H67H=); (G) c.352_363delGCGTTTGACTTA (A118_L121del, Frameshift); (H)c.1058A>G(p.Y353C); (I)c.1103_1104insG [(Frameshift and stop at codon 377(TGA)]; (J) c.1242-1245delAGAC [(L414_D415del, Framashift and stop at codon 434(TAA)]. **Figure S4.** SMAD4 Y353C has no effects to cell proliferation in vitro. (A, B) The proliferation ability of the SW1990 and PANC-1 cell line was detected and the results showed that there was no significant difference between the negative control group (NC), SMAD4 wt group and SMAD4 Y353C group. All data are shown as mean ± SD of 3 independent experiments performed in triplicate (one-way ANOVA, *p>0.05*).

## Data Availability

The datasets obtained or analysed during the current study are available from the corresponding author upon reasonable request.

## Abbreviations

AJCC: American Joint Committee on Cancer
CSC: Cancer stem cell
DPC4: Pancreatic cancer deletion gene4
EMT: Epithelial-mesenchymal-transition
HRP: Horseradish peroxidase
OS: Overall survival
PDAC: Pancreatic ductal adenocarcinoma
PMSF: Phenylmethanesulfonyl fluoride
PVDF: Polyvinylidene fluoride
qRT-PCR: Real-time quantitative polymerase chain reaction
