# Supplementary material for: SMAD4 Y353C promotes the progression of PDAC
Source: BMC Cancer. 2019 Nov 4;19:1037. doi: 10.1186/s12885-019-6251-7 (PMC6829834; doi:10.1186/s12885-019-6251-7)
Supplement: Supplementary file 1 — Additional file 1: Figure S1. The whole exons of SMAD4 gene primers for details. Figure S2. SMAD4 gene 1~11 exons’ PCR products electrophoresis results. Figure S3. SMAD4 mutation analysis. (A) c.6C>T (p.D2D=); (B) c.29C>T (p.P10L); (C) c.34A>G (p.S12G); (D) c.112A>G (p.R38G); (E) c.153_d3elA [Frameshift, stop at codon 57(TAA)]; (F) c.201 T>C (p.H67H=); (G) c.352_363delGCGTTTGACTTA (A118_L121del, Frameshift); (H)c.1058A>G(p.Y353C); (I)c.1103_1104insG [(Frameshift and stop at codon 377(TGA)]; (J) c.1242-1245delAGAC [(L414_D415del, Framashift and stop at codon 434(TAA)]. Figure S4. SMAD4 Y353C has no effects to cell proliferation in vitro. (A, B) The proliferation ability of the SW1990 and PANC-1 cell line was detected and the results showed that there was no significant difference between the negative control group (NC), SMAD4 wt group and SMAD4 Y353C group. All data are shown as mean ± SD of 3 independent experiments performed in triplicate (one-way ANOVA, p>0.05). [file 12885_2019_6251_MOESM1_ESM.doc]

**Electronic supplementary material**

| EXON | Forward primer | reverse primer | Amplification length（bp） | annealing temperature（℃） |
| --- | --- | --- | --- | --- |
| SMAD4-1 | CCTGATAGGCCATGGGTGAGT | GCTTGAAAGGAAACGTAGCAAGTT | 558 | 56 |
| SMAD4-2 | TGGTAGGATTGTGAGGATTAAATCAG | CGCGGGCTATCTTCCAAAT | 368 | 56 |
| SMAD4-3 | TTGATATTTTGCCCCTTTAGAACAT | TGCCGCTCACACAAACTAATTC | 350 | 60 |
| SMAD4-4 | GTTTATCAAGAAACTGAGGAGTACCTTTT | TGCCGCTCACACAAACTAATTC | 600 | 58 |
| SMAD4-5 | CCTGATAGGCCATGGGTGAGT | TAAGGCCCACATGGGTTAATTT | 350 | 58 |
| SMAD4-6 | AAGGACTGTTGCAGATAGCATCAG | ACAGAAAACAAAGCCCTACCAAAA | 361 | 54 |
| SMAD4-7 | CTTGGCAGATAGCACTGAAATGTTAG | AAAGCCTGTGTTTGTGCGTTT | 350 | 56 |
| SMAD4-8 | TCCCCTCCCTTTACCCTTTCT | GATGGAGTGCTTACAAATGTT | 450 | 59 |
| SMAD4-9 | ACATGCTCCTGACACATAGTAAGTGTT | CCCAGATTTCAATTCTTTTGACAA | 462 | 58 |
| SMAD4-10 | TGAGTTTTAAATAAGTCAGGCATTGG | TTCAAAAATGTCATCATCCCAGTAA | 358 | 58 |
| SMAD4-11 | CCTTAACCAAAAGTGTGCAGCTT | TTGTAGTCCACCATCCTGATAAGGT | 450 | 56 |

**Figure S1.**The whole exons of SMAD4 gene primers for details


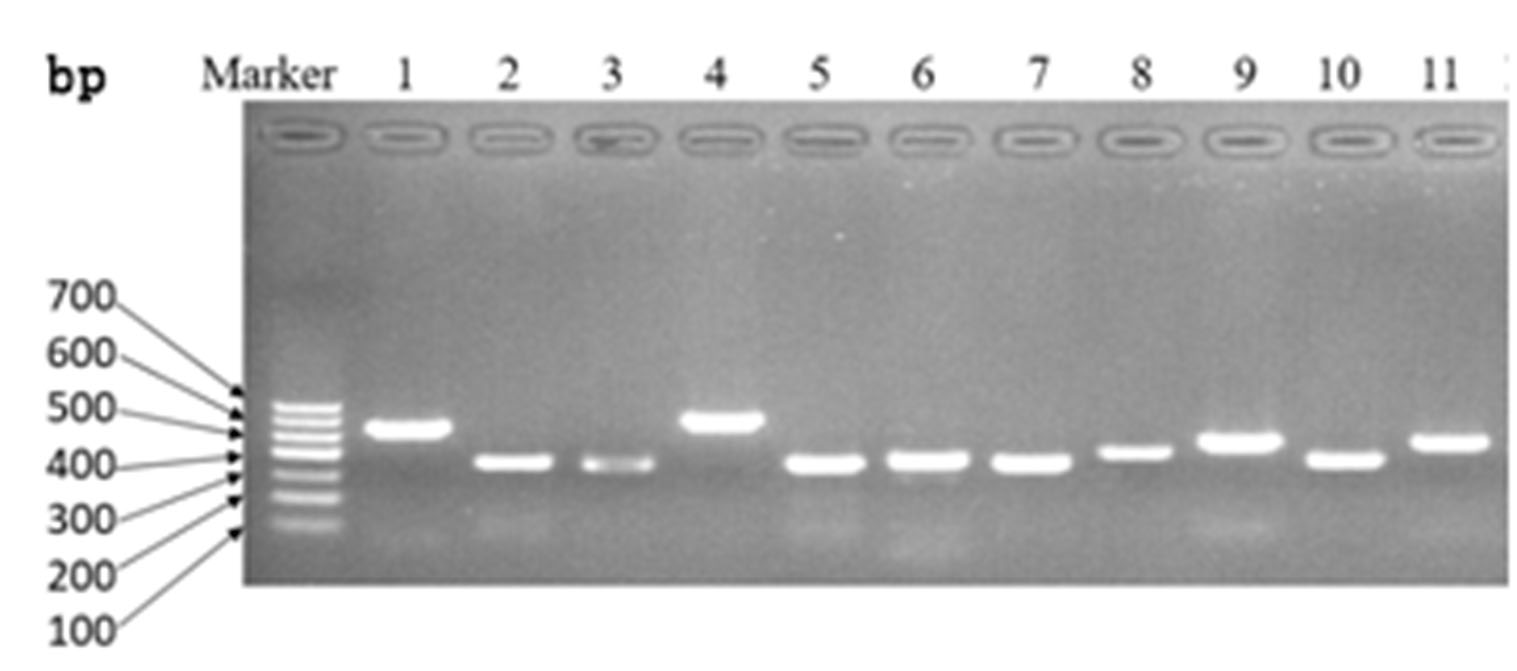


**Figure S2.** SMAD4 gene 1～11 exons’ PCR products electrophoresis results.

**
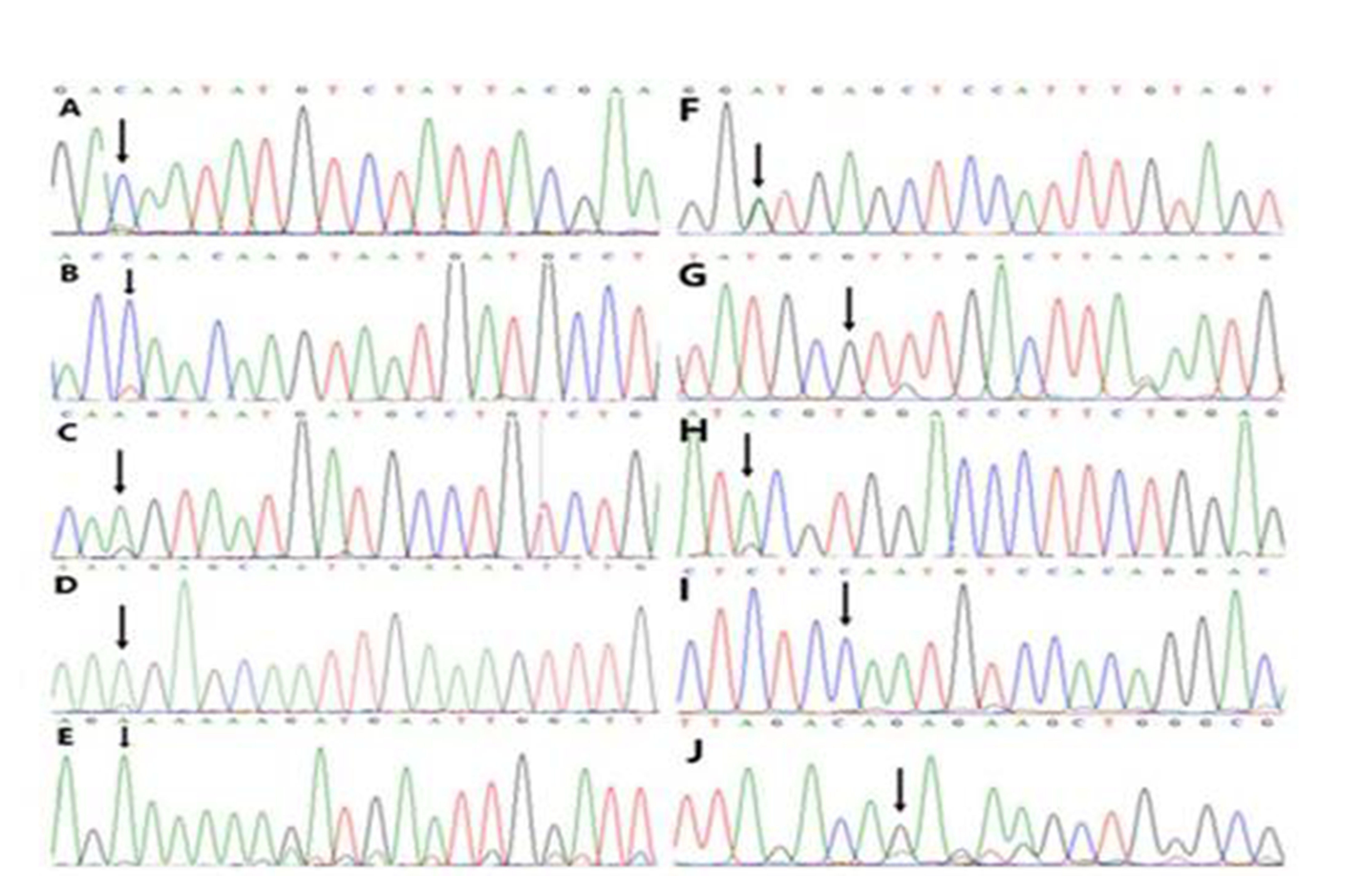
**

**Figure S3.SMAD4 mutation analysis.** (A) c.6C＞T ( p.D2D=); ( B) c.29C＞T (p.P10L); ( C) c.34A＞G (p.S12G); (D) c.112A＞G (p.R38G); (E) c.153_d3elA [Frameshift ,stop at codon 57(TAA)]; (F)c.201T＞C (p.H67H=); (G) c.352_363delGCGTTTGACTTA (A118_L121del,Frameshift); (H)c.1058A＞G(p.Y353C);(I)c.1103_1104insG [(Frameshift and stop at codon 377(TGA)]; (J) c.1242-1245delAGAC [(L414_D415del, Framashift and stop at codon 434(TAA)].

**
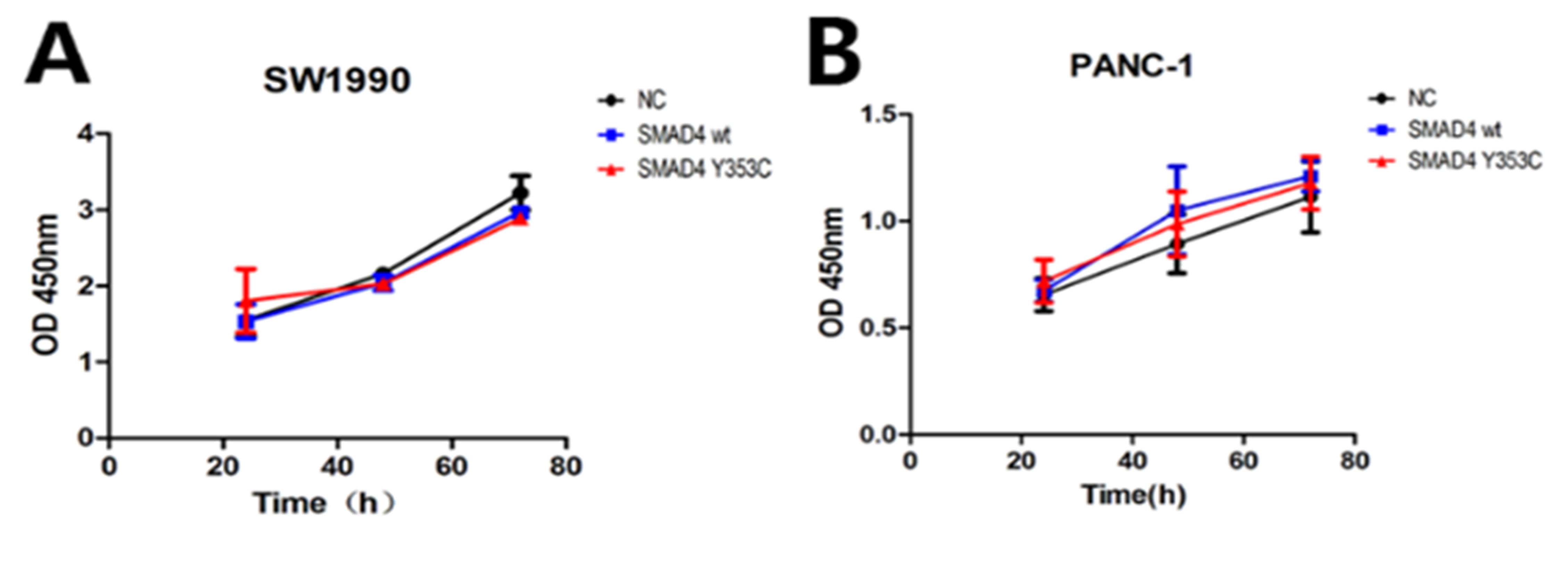
**

**Figure S4. SM****AD4 Y353C has no effects to cell proliferation in vitro.**(A,B) The proliferation ability of the SW1990 and PANC-1 cell line was detected and the results showed that there was no significant difference between the negative control group (NC), SMAD4 wt group and SMAD4 Y353C group. All data are shown as mean ± SD of 3 independent experiments performed in triplicate (one-way ANOVA, *p＞0.05*).
